# Definitive outcomes in patients with rifampicin-resistant tuberculosis treated in Niger from 2012 to 2019: A retrospective cohort study

**DOI:** 10.1093/inthealth/ihac016

**Published:** 2022-04-14

**Authors:** Mahamadou Bassirou Souleymane, Tom Decroo, Saïdou Mamadou, Alphazazi Soumana, Ibrahim Mamane Lawan, Assiatou Gagara-Issoufou, Eric Adehossi, Nimer Ortuño-Gutiérrez, Lutgarde Lynen, Leen Rigouts, Bouke Catherine de Jong, Armand Van Deun, Alberto Piubello

**Affiliations:** Damien Foun dation Niger, TB-MR, BP: 1065 Niamey, Niger; Institute of Tropical Medicine, Unit of Mycobacteriology, Nationalestraat 155, 2000 Antwerp, Belgium; Research Foundation Flanders, 1000 Brussels, Belgium; Université Abdou Moumouni de Niamey, Faculté des Sciences de la Santé, BP: 237 Niamey, Niger; Programme National de Lutte contre la Tuberculose, Coordination, BP: 613 Niamey, Niger; Damien Foun dation Niger, TB-MR, BP: 1065 Niamey, Niger; Université Abdou Moumouni de Niamey, Faculté des Sciences de la Santé, BP: 237 Niamey, Niger; Université Abdou Moumouni de Niamey, Faculté des Sciences de la Santé, BP: 237 Niamey, Niger; Damien Foundation Brussels, TB-MR, Boulevard Léopold II 263, 1081 Brussels, Belgium; Institute of Tropical Medicine, Unit of Mycobacteriology, Nationalestraat 155, 2000 Antwerp, Belgium; Institute of Tropical Medicine, Unit of Mycobacteriology, Nationalestraat 155, 2000 Antwerp, Belgium; University of Antwerp, Biomedical Sciences, Prinsstraat 13, 2000 Antwerp, Belgium; Institute of Tropical Medicine, Unit of Mycobacteriology, Nationalestraat 155, 2000 Antwerp, Belgium; Independent Consultant, 3000 Leuven, Belgium; Damien Foun dation Niger, TB-MR, BP: 1065 Niamey, Niger; Damien Foundation Brussels, TB-MR, Boulevard Léopold II 263, 1081 Brussels, Belgium

**Keywords:** bedaquiline, Niger, rifampicin-resistant, short treatment regimen, TB

## Abstract

**Background:**

Outcomes of retreatment for rifampicin-resistant tuberculosis (RR-TB) are rarely reported. We report ‘definitive outcomes’ after a cascade approach to RR-TB treatment. After a bacteriologically adverse outcome for the 9-months fluoroquinolone-based Short Treatment Regimen (STR), patients were retreated with a bedaquiline-based regimen (BDQ-regimen).

**Methods:**

A Retrospective cohort study of RR-TB patients treated with the STR during 2012–2019 and retreated with a BDQ-regimen in case of failure or relapse was conducted. Definitive relapse-free cure took into account BDQ-regimen outcomes.

**Results:**

Of 367 patients treated with the STR, 20 (5.4%) experienced failure or relapse. Out of these 20 patients, 14 started a BDQ-regimen, of whom none experienced failure or relapse. Definitive end of treatment outcomes of STR after revising with third-line BDQ-regimen outcomes, 84.7% (311/367) were cured relapse-free, 10.6% (39/367) died during treatment and 3.0% (11/367) were lost to follow-up during treatment with either the STR or BDQ-regimen. Six patients (1.6%; 6/367) with STR failure/relapse died before starting a BDQ-regimen. No patient had definitive treatment failure or relapse and remained without treatment.

**Conclusions:**

If fluoroquinolone resistance is excluded or rare, it is beneficial to use fluoroquinolone as the core drug for a first RR-TB treatment regimen and to safeguard bedaquiline for those in need of retreatment.

## Introduction

Globally, <60% of patients with rifampicin resistant-TB (RR-TB) are treated successfully.^1^ In 2016, the 9-mo fluoroquinolone-based Short Treatment Regimen (STR) was endorsed by the WHO for patients with RR-TB.^2^ High-dose fluoroquinolone (FQ) was used as the core drug, a drug with both high bactericidal and sterilising activity, thus driving the efficacy of the regimen. A second-line injectable drug (SLI) was used to protect against acquired FQ resistance. With this regimen treatment, success was 80% or higher.^3,4^ Studies show the proportion of treatment success among patients treated for RR-TB. Whereas treatment success among patients with RR-TB is often reported, data on managing patients experiencing failure or relapse after RR-TB treatment are scarce. It remains unclear whether, following an unfavourable RR-TB treatment outcome, a sufficient number of effective TB drugs remain to constitute a robust RR-TB retreatment regimen and whether TB can finally be cured or not.

In its 2020 guidelines, the WHO recommends using all most powerful second-line anti-TB drugs together in one all-oral regimen. This all-oral regimen includes both second-line core drugs, FQ and bedaquiline.^5^ How to treat those with an adverse outcome and a high probability of acquired resistance to both FQ and bedaquiline remains unclear. Moreover, most RR-TB studies do not show retreatment outcomes for those with treatment failure or relapse.

In Niger, in Damien Foundation-supported RR-TB clinics, rather than using a one­shot approach with both second-line core drugs in a single RR-TB regimen, a ‘cascade of regimens’ approach was used.^6^ FQ served as the core drug for the second-line STR and bedaquiline served as the core drug to constitute a third-line bedaquiline-based treatment regimen (BDQ-regimen) for those treated unsuccessfully with the STR. This ‘cascade of regimens’ approach has not yet been evaluated for RR-TB. We therefore report ‘definitive outcomes’, revising bacteriologically adverse second-line STR outcomes (treatment failure or relapse) with third-line outcomes after retreatment with a BDQ-regimen. Definitive relapse-free cure (either cure or treatment completion without proof of relapse) was calculated among all those who started a second-line STR in Niger during 2012–2019. Treatment monitoring included 12-mo post-treatment follow-up to identify relapse.

## Methods

This was a retrospective cohort study of definitive outcomes among all RR-TB patients treated with the STR during 2012–2019 at all programmatic management drug-resistant TB units in Niger. Follow-up data were collected until June 2021.

Culture on solid medium and susceptibility testing to diagnose RR-TB were initially performed in the National TB laboratory in Niamey. Xpert MTB/RIF (Xpert; Cepheid, Sunnyvale, CA, USA) testing was introduced in 2013; Second-Line Line Probe Assay (SL-LPA; GenoType MTBDRsl; Hain Lifesciences, Nehren, Germany) testing became available in January 2016. In addition, pretreatment samples in cetylpyridinium chloride were systematically referred to the supra-national TB reference laboratory of the Institute of Tropical Medicine in Antwerp for culture to à posteriori determine the initial resistance profile (with too long a turnaround time to inform clinical decision-making). The study setting and procedures are published elsewhere.^4,7^

The 9–11 mo STR was the standard treatment regimen and included a FQ (moxifloxacin or gatifloxacin), clofazimine, ethambutol and pyrazinamide throughout, supplemented by kanamycin, prothionamide and high-dose isoniazid (10 mg/kg) during the first 4 mo (6 mo if conversion to smear microscopy was delayed). Since 2017, the second-line injectable kanamycin has been replaced by linezolid in case of any hearing disturbance on audiometry.^7^

In case of failure or relapse, patients started a BDQ-regimen, including bedaquiline throughout treatment and, depending on drug susceptibility testing (DST) results, linezolid with either a second-line injectable or delamanid. The STR structure was respected. The BDQ-regimens used bedaquiline as the core drug, and companion drugs added either bactericidal or sterilising activity.^8^

To determine a definitive relapse-free cure, bacteriologically adverse outcomes were revised taking into account STR and BDQ-regimens, with 1-y post-treatment follow-up for both regimens.

The National Agency for the Research on AIDS and hepatitis (ANRS) scales were used to grade adverse events (grade 1: mild; grade 2: moderate; grade 3: severe; grade 4: life-threatening or permanently disabling).^9^

## Results

**Figure 1.**
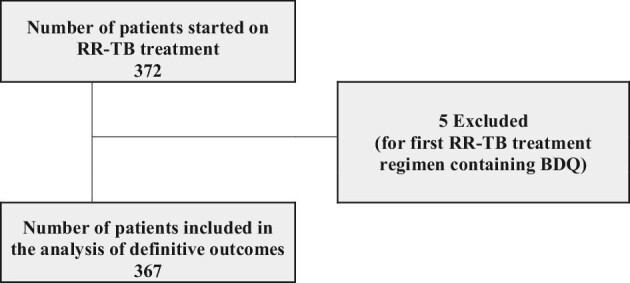
Flowchart of the study population. BDQ, bedaquiline; RR-TB, rifampicin-resistant TB.

Of 372 patients treated for RR-TB, 367 received the STR and were included in the analysis. Five patients, because of contraindication to the second-line injectables and linezolid, were directly treated with a BDQ-regimen and excluded from the analysis (Figure 1). These five patients were cured relapse-free.

Of 367 STR patients, 93,7% had TB susceptible to both FQ and SLI (Table 1). Most (320) were treated with kanamycin throughout the intensive phase. Following audiometry, in 47 (13.0%) patients kanamycin was replaced by linezolid, either during or at the start of the intensive phase, which prevented severe hearing loss in all except two patients.^7^

**Table 1.** Initial resistance to FQ and second-line injectables among rifampicin-resistant patients treated with a short treatment regimen during 2012–2019 in Niger

Number of patients enrolled on a first RR-TB treatment regimen	367	
No DST results for FQ and SLI	64	17.4%
DST results for FQ and SLI available	303	82.6%
*Of which:*		
Susceptible to FQ and SLI	284	93.7%
Susceptible to FQ and resistant to SLI	1	0.3%
Resistant to FQ and SLI	0	0%
Resistant to FQ and susceptible to SLI	18	5.9%
*Of which:*		
High-level FQ resistance	8	
Low-level FQ resistance	10	

Abbreviations: DST, drug susceptibility testing; FQ, fluoroquinolone; RR-TB, rifampicin-resistant TB; SLI, second-line injectable.

End-of-treatment STR outcomes were treatment success (82.3%; 302/367), death (10.6%; 39/367), loss to follow-up (3.0%; 11/367) and treatment failure (4.1%; 15/367). Of 302 successfully treated patients, 86.8% (262) and 77.8% (235) had negative sputum smear and/or culture results at 6 and 12 mo post-treatment, respectively. Of 302 successfully treated patients, five (1.7%) experienced relapse. Sixty-two (20.5% of 302) patients were without a 12-mo post-treatment ‘bacteriological result’: eight died (four smear and/or culture negative at 6 mo post-treatment), 28 (10 smear and/or culture negative at 6 mo) refused to come to the clinic but were alive and reportedly healthy and, for the other 26, post-treatment follow-up was lacking: 16 could not be contacted by telephone, three had moved to another country and seven were not yet eligible for the 12-mo post-treatment evaluation (Table 2).

**Table 2.** Definitive outcomes among patients treated with a short rifampicin-resistant TB treatment regimen during 2012–2019 in Niger

STR treatment outcomes (N=367)		
Cured/completed treatment	302	82.3%
Died	39	10.6%
Lost to follow-up	11	3%
Failure	15	4.1%
STR post-treatment follow-up (N=302)		
Culture (or smear)-negative at 6 mo	262	86.8%
Culture (or smear)-negative at 12 mo	235	77.8%
Relapse	5	1.7%
Death at 12 mo	8	2.8%
Refused at least one evaluation but alive at 12 mo	28	9.3%
Lost to follow-up at 12 mo	16	5.3%
Moved to another country	3	1%
Awaiting smear and/or culture before the end of 12 mo	7	2.3%
Of patients with STR failure/relapse (N=20)	20	
Died in between treatments	6	30%
Enrolled on a BDQ-regimen	14	70%
BDQ-regimen treatment outcomes (N=14)		
Cured/completed treatment	14	100%
Died	0	
Lost to follow-up	0	
Failure	0	
BDQ-regimen post-treatment follow-up (N=14)		
Culture (or smear)-negative at 6 mo	13	92.9%
Culture (or smear)-negative at 12 mo	10	71.4%
Relapse	0	0%
Death at 12 mo	1	7.1%
Refused at least one evaluation but alive at 12 mo	2	14.3%
Lost to follow-up at 12 mo	0	0%
Awaiting smear and/or culture before the end of 12 mo	1	7.1%
Definitive treatment outcomes STR (N=367)		
Cured/completed treatment^#^	311	84.7%
Died during treatment^#^	39	10.6%
Lost to follow-up during treatment^#^	11	3.0%
Failure/relapse during STR, then died before starting a BDQ-regimen	6	1.6%
Failure/relapse without treatment option	0	0.0%

Abbreviations: BDQ-regimen, bedaquiline regimen; STR, short rifampicin-resistant TB treatment regimen.

^#^either STR or BDQ-regimen.

Of 20 patients with failure or relapse after treatment with the STR, six (all with previous STR failure) died before starting a BDQ-regimen. The time from treatment failure to death ranged from 2 wk to 2 mo. Fourteen started a BDQ-regimen. The regimen systematically included linezolid plus amikacin or delamanid plus three or four companion drugs according to DST results, as shown in Table 3. Culture conversion occurred in nine patients at 1 mo and in the others during 2–5 mo. All 14 were cured and none experienced relapse but one died before the 6-mo post-treatment evaluation. Post-treatment follow-up was not complete for one patient.

**Table 3.** Resistance profile and outcomes of 20 patients with short treatment regimen failure or relapse and retreated with a bedaquiline-based regimen in Niger, during 2012–2019

	Drugs for which susceptibility testing was performed at initiation of STR (in bold if susceptible, underscored if low-level resistance)	Conversion (mo if converted)	Outcome	Drugs for which resistance was detected at recurrence (underscored shows if acquired^#^)	BDQ-regimen* (drugs proven to be susceptible in bold)	BDQ-regimen: total treatment duration (mo)	Conversion (mo if converted)	Outcome (CU, D, FL or RL)	Adverse events (grade)
1	4**K**PHCGZE/5CGZE	3	FL	RHESO2Eto	NA	NA	NA	D	NA
2	6**K**PHC**M**ZE/5C**M**ZE	No	FL	RHESO2O8Eto	NA	NA	NA	D	NA
3	6**K**PHCMZE/5CMZE	No	FL	RHESO2O8Eto	NA	NA	NA	D	NA
4	6**KP**HCMZE/5CMZE	No	FL	RHESKO2O8Eto	NA	NA	NA	D	NA
5	6**K**PHCMZE/5CMZE	No	FL	RHESKO2O8Eto	NA	NA	NA	D	NA
6	5KPHCMZE/5CMZE	2	FL	RO2O8	NA	NA	NA	D	NA
7	4**K**PHCGZE/5CGZE	3	RL	RHESO2Eto	**CmBdqLzd**PASCLfx	20	5	CU	PN(2); L(1); H(1); HL(2); R(1); SP(3)
8	4**K**PHCGZE/5CGZE	No	FL	RHESO2O8Eto	**AmBdqLzd**PASCCs	20	3	CU	PN(2); H(2); HL(3)
9	6**K**PHCMZE/5CMZE	No	FL	RHESO2O8Eto	**AmBdqLzd**PASCLfx	15	3	CU	L(1); H(1); R(1)
10	4**K**PHC**M**ZE/5C**M**ZE	1	RL	RHESO2O8Eto	**AmBdqLzd**PASCLfx	15	1	CU	PN(2); L(2); H(2); HL(1)
11	4**K**PHCMZE/5CMZE	1	RL	RHESO2Eto	**AmBdqLzd**PASCLfx	15	1	CU	PN(2); L(2); H(1); HL(2)
12	4**KP**HCMZE/5CMZE	1	RL	RHESO2O8	**AmBdqLzd**PASCHZ	12	1	CU	PN(1); H(1); HL(1); R(2); GI(1); QT(2); VI(1)
13	4**KP**HC**M**ZE/5C**M**ZE	No	FL	RHESO2O8	**AmBdqLzd**PASCHZ	12	2	CU	L(1); H(1); HL(1); A(3)
14	4KPHCMZE/5CMZE	6	FL	RO2	**AmDlmBdqLzd**CHZ	12	4	CU	PN(2); L(2); H(1); HL(3); A(2)
15	4**K**PHC**M**ZE/5C**M**ZE	No	FL	RHESKO2O8Eto	**DlmBdqLzd**CHZ	12	1	CU	PN(3); L(1); H(1)
16	4**K**PHC**M**ZE/5C**M**ZE	2	RL	R**H**ESO2O8Eto	**DlmBdqLzd**CHZ	12	1	CU	PN(2); L(1); A(1)
17	4**K**PHCMZE/5CMZE	2	FL	R**H**ESO2O8Eto	**DlmBdqLzd**CHZ	12	1	CU	PN(1); A(3); R(1)
18	5**K**PHC**M**Z**E**/5C**M**Z**E**	2	FL	R**H**SKEto	**DlmBdqLzd**CHZ	12	1	CU	PN(2); A(2)
19	4**K**PHCMZE/5CMZE	No	FL	R**H**ESO2O8Eto	**DlmBdqLzd**CHZ	12	1	CU	PN(2); L(1); H(2); A(4); GI(2); SP(1); QT(1)
20	4**KPH**CMZ**E**/5CMZ**E**	2	FL	RSO2O8	**DlmBdqLzd**CHZ	12	1	CU	PN(2); L(3); A(2); GI(2)

Abbreviations:

Drugs: Am, Amikacin; Bdq, Bedaquiline; C, Clofazimine; Cm, Capreomycin; Cs, Cycloserine; Dlm, Delamanid; E, Ethambutol; Eto, Ethionamide; H, Isoniazid; K, Kanamycin; Lfx, Levofloxacin; Lzd, Linezolid; M, Moxifloxacin; O2, Ofloxacine low level resistance; O8, Ofloxacine high-level resistance; P, Prothionamid, PAS, para-aminosalicylic acid, R, Rifampicin; S, Streptomycin; Z, Pyrazinamid; NA, not applicable.

Outcomes: CU, Cured; D, Death; FL, Failure; RL, Relapse.

Adverse events and grade: A, Anaemia; GI, Gastrointestinal disorder; H, Hepatitis; HL, Hearing loss; L, Hyperlipasemia; PN, Peripheral neuritis; QT, QTc prolongation; R, Renal failure; SP, Skin pigmentation; VI, Visual impairment; 1, Adverse event Grade 1; 2, Adverse event Grade 2; 3, Adverse event Grade 3; 4, Adverse event Grade 4.

# acquired resistance: resistance not present at baseline of the FQ-based STR, but present on the recurrence sample after FQ-based STR.

*When phenotypic DST results were available (which was not always the case) before starting the BDQ-regimen and showed low-level FQ resistance or FQ susceptibility, a FQ was added to the regimen, but without counting on it as the regimen's core drug.

All patients experienced at least two adverse events during treatment with the BDQ-regimen. Overall, 58 adverse events were reported; 57.1% (8/14) of patients had four or more adverse events. The most common adverse event was peripheral neuritis, which occurred in 12 of 14 patients. The majority (86.2%, 50 out of 58) of adverse events were grade 1 or 2, seven were grade 3 and one was grade 4 (Table 4).

**Table 4.** Adverse events grading for patients treated with a bedaquiline-based regimen

	Grade 1^#^		Grade 2^#^		Grade 3^#^		Grade 4^#^		Total *	
Adverse events	n	%	n	%	n	%	n	%	n	%
Peripheral neuritis	2	18.2%	9	75.0%	1	8.3%	0	0.0%	12	20.7%
Hyperlipasemia	6	60.0%	3	30.0%	1	10.0%	0	0.0%	10	17.2%
Hepatitis	7	70.0%	3	30.0%	0	0.0%	0	0.0%	10	17.2%
Hearing loss	3	42.9%	2	28.6%	2	28.6%	0	0.0%	7	12.1%
Anaemia	1	14.3%	3	42.9%	2	28.6%	1	14.3%	7	12.1%
Renal failure	3	75.0%	1	25.0%	0	0.0%	0	0.0%	4	6.9%
Gastrointestinal disorder (nausea and vomiting)	1	33.3%	2	66.7%	0	0.0%	0	0.0%	3	5.2%
Skin pigmentation	1	50.0%	0	0.0%	1	50.0%	0	0.0%	2	3.4%
QTc prolongation	1	50.0%	1	50.0%	0	0.0%	0	0.0%	2	3.4%
Visual impairment	1	100.0%	0	0.0%	0	0.0%	0	0.0%	1	1.7%
Total	26	44.8%	24	41.4%	7	12.1%	1	1.7%	58	100.0%

# percentage by grade for each adverse event.

*percentage of all adverse events.

After revising bacteriologically adverse STR outcomes taking into account BDQ-regimen outcomes, 84.7% (311/367) were cured or completed treatment, 10.6% (39/367) died during treatment, 3.0% (11/367) were lost to follow-up during treatment with either the STR or BDQ-regimen, while 1.6% (6/367) experienced failure/relapse during the STR and died before starting a BDQ-regimen. No patient had definitive treatment failure or relapse and remained without treatment option.

## Discussion

Using a cascade of regimens approach, relying on a BDQ-regimen for those treated unsuccessfully with a STR resulted in a successful definitive outcome in 84.7% of patients. Most patients converted at 1 mo. The injectable and/or linezolid in the BDQ-regimen contributed to assure high early bactericidal activity and prevent resistance development.^8^ The contribution of delamanid to the regimen's bactericidal activity has to be confirmed.^10^ With this cascade of regimens approach, no patient was left without a treatment option. Successful treatment outcomes for patients with FQ-resistant RR-TB have been reported elsewhere.^11^ Success was strongly dependent on whether the regimen's core drug was effective and well protected.

To the best of our knowledge, this is the first study to report and describe in detail definitive outcomes for RR-TB patients treated with the STR, followed by the BDQ-regimen in case of a bacteriologically adverse outcome. Most studies report end-of-treatment results without post-treatment follow-up showing relapse rates. It is even more rare to find data on the composition and outcome of retreatment regimen for patients who experienced a bacteriologically unfavourable RR-TB outcome.^12^

So far only a few core drugs have been identified: rifampicin for first-line, and FQ and bedaquiline for second-line treatment. The ‘cascade of regimens’ approach implies that each regimen relies on a single core drug, a drug with both high bactericidal and sterilising activity that drives the efficacy of the regimen.^8^ Using a single core drug per regimen, treatment options remain available for those treated unsuccessfully. However, when RR-TB is diagnosed, the WHO recommends combining both second-line core drugs (FQ and bedaquiline) in a single regimen.^5^ This WHO-recommended short oral bedaquiline-containing treatment regimen showed 71% (815/1140 with outcomes reported) therapeutic success in a recent study from South Africa, considerably lower than the 83.1% success obtained with the STR in Niger during the last 10 y.^4,13^ In the South African cohort culture did not convert in 12.8% (253/1962 with conversion data reported) of patients. The proportion with failure or relapse was not explicitly reported. The same study found 3.8% baseline bedaquiline resistance in patients with RR-TB, and 2.3% acquired bedaquiline resistance during treatment with this currently recommended all-oral treatment regimen.^13^ Similar findings were reported by Nimmo et al. in 2020.^14^ In both studies bedaquiline resistance was often accompanied by resistance to FQ. How to treat those with failure or relapse, thus with a high probability of RR-TB also resistant to FQ and bedaquiline, is not explained. They will not be eligible for the Nix regimen (bedaquiline, pretomanid and linezolid).^15^ Salvage regimens, without the active core drug, result in less than 55% success.^1^ In the absence of highly effective treatment options for people infected with such an extensively drug-resistant strain of TB, TB resistant to the two core second-line drugs, FQ and bedaquiline, will spread.

The frequency of adverse events experienced by patients treated with the linezolid-containing BDQ-regimen is coherent with what was reported during the Nix trial, evaluating the efficacy and safety of the combination of bedaquiline and linezolid with pretomanid. In this trial all patients experienced at least one adverse event and 57% had a grade 3 (or higher) adverse event, mostly due to the use of linezolid.^14^ In a previous study we showed that a short regimen with linezolid resulted in more grade 3 adverse events than the same short regimen using a second-line injectable drug as companion drug with resistance-prevention activity.^7^

The limitations of this study are inherent to its design. There was no control group. Only in a limited number of patients could the definitive outcome not be bacteriologically assessed. Data were collected prospectively and were complete. Moreover, data from this nationwide cohort spanning a long period represents the reality of Niger and may also be generalisable to other settings with a well-organised national TB programme and a relatively low prevalence of resistance to FQs, thus with only a minority of patients in need of third-line RR-TB treatment.

In conclusion, to be successful, TB treatment regimens require a core drug. In Niger, not a single RR-TB patient remained infectious with bacilli showing amplified resistance. From the perspective of TB control and the patient's right to receive the most effective approach to treatment, in Niger it was beneficial to use FQ as the core drug for a first RR-TB treatment regimen and to safeguard bedaquiline for those in need of a retreatment RR-TB treatment regimen.

## Data Availability

The authors have permission to publish the data shown in the manuscript. In order to have access to the data, please request permission from the National Tuberculosis Program of Niger (Dr Soumana Alphazazi via E-mail: s_alphazazi@yahoo.fr) and Damien Foundation Niger (Dr Mahamadou Bassirou Souleymane via E-mail: bachirsoul@gmail.com).
